# Spontaneous rupture of the urinary bladder caused by eosinophilic cystitis in a male after binge drinking: A case report

**DOI:** 10.1097/MD.0000000000009170

**Published:** 2017-12-22

**Authors:** Xiaowen Zhang, Guangyuan Zhang, Lei Zhang, Chao Sun, Ning Liu, Ming Chen

**Affiliations:** a School of Medicine, Southeast University; b Department of Urology Surgery, Zhongda Hospital, Southeast University, Nanjing, China.

**Keywords:** eosinophilic cystitis, spontaneous rupture, urinary bladder

## Abstract

**Introduction:**

Spontaneous rupture of the urinary bladder is a rare, difficult to diagnose surgical emergency with a high mortality, there are many causes for spontaneous rupture of the urinary bladder, but we only found 2 reports on this condition in our literature search. A 36-year-old male patient was admitted with “whole abdominal pain associated with hematuria for 5 hours.” Our patient did not have a history of definite allergy, but a long-term history of alcohol abuse. This patient was followed up for 1 year, and the cystoscopy recheck showed that the bladder lesion had healed.

**Conclusions:**

Since eosinophilic cystitis is associated with long-term alcohol consumption, we recommended that the patient should stop drinking and taking antihistamines.

## 1 Introduction

Spontaneous rupture of the urinary bladder is a rare, difficult to diagnose surgical emergency with a high mortality.^[1]^ There are many causes for spontaneous rupture of the urinary bladder, but we only found 2 reports on this condition in our literature search.^[2,3]^ Moreover, spontaneous rupture of the urinary bladder caused by eosinophilic cystitis after binge drinking has not yet been reported. Our department had recently admitted one such case, which we report here along with a literature review.

## 2 Clinical data

**Figure 1:**
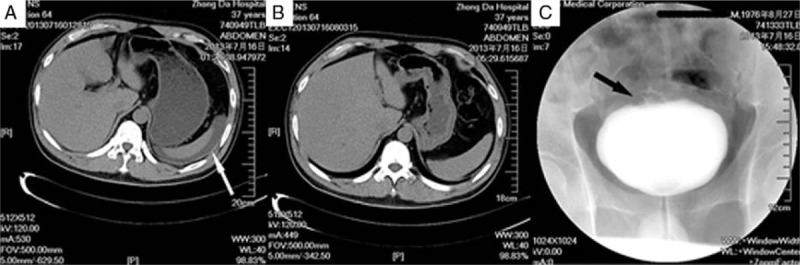
Imaging examination of the patient. (A) Axial computed tomography (CT) image showed a few of liquid dark areas around the spleen (white arrow); (B) CT recheck indicated the disappearance of the intraperitoneal fluids; and (C) cystography indicated a small amount of suspicious contrast agent leakage (black arrow).

A 36-year-old male patient was admitted with “whole abdominal pain associated with hematuria for 5 hours.” There were no urinary symptoms in recent 3 months before. Five hours prior to onset, he was in a semidrunken state due to binge drinking and complained of whole abdominal pain associated with inability to urinate. Physical examination indicated that he was in a state of obnubilation, showing a painful appearance, with stable vital signs; his abdomen was slightly elevated, with tenderness and rebound tenderness in the whole abdomen; and filled and bulged bladder with obvious tenderness was found in the lower abdomen above the pubic region. Auxiliary examination indicated normal blood liver function, electrolytes, and amylase, creatinine 145 mmol/L, WBC 13.9 × 10^9^/L, neutrophils 90%, RBC 159 g/L, and Plt 150 × 10^9^/L. Unenhanced computed tomography (CT) scanning indicated no free gas below the bilateral diaphragm, some liquid dark areas around the spleen and subanterior gap of the liver (Fig. 1A), well-filled bladder in the lower abdomen, and thickened intima at the top bladder with surrounding exudation. Pale red liquid was withdrawn during abdominocentesis. Considering the possibility of subintraperitoneal bladder rupture, urethral catheterization was applied. After removal of a large volume of bloody urine (with the color resembling watermelon juice), the patient's pain was greatly relieved. Six hours later, all the symptoms had disappeared. CT recheck indicated the disappearance of intraperitoneal fluids (Fig. 1B). Cystography on the following day indicated a small volume of suspicious contrast agent leakage (Fig. 1C), which further confirmed the diagnosis of spontaneous rupture of the urinary bladder.

**Figure 2:**
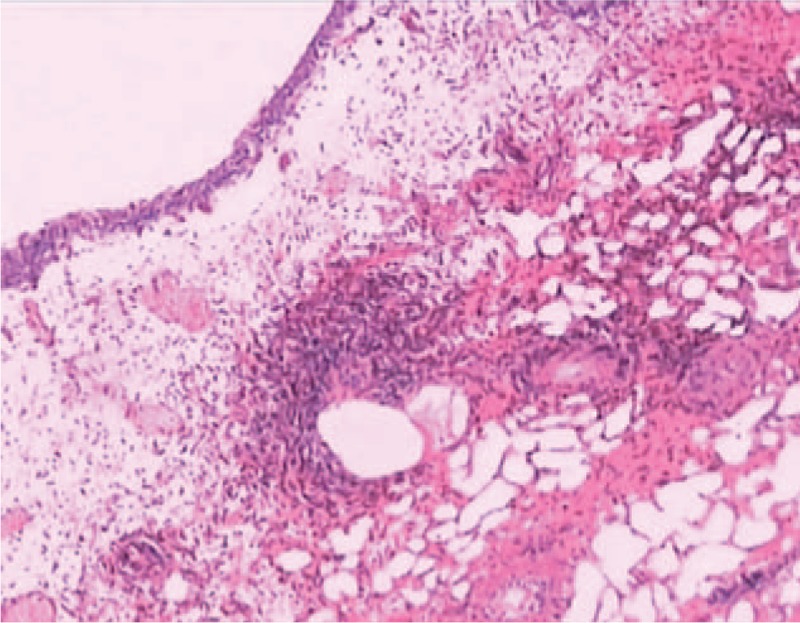
Eosinophilic cystitis was confirmed histologically (20×, hematoxylin and eosin stain [H&E]).

After admission, urethral catheterization was continued to ensure emptying of the bladder, along with symptomatic intravenous administration of ceftriaxone. A week later, urethral cystoscopy indicated several, scattered, strawberry-like bulges associated with surface ulcers and edematous thickening of the surrounding tissues, both at the bottom and top of the bladder. Biospy was carried out at top of the bladder which was suspected ruptured site. Histopathological examination revealed eosinophilic cystitis lesions (Fig. 2). One-hundred milligram lavo-ofloxacin was given intraveinously, 100 mg celecoxib and 10 mg loratadine were given orally everyday postoperatively. The patient became healthy and retained good prognosis during the following half-year follow-up. Cystoscope was performed at 6 months later, which showed the bladder lesion had healed. Patient consent was obtained. Ethics approval was not required for this paper as it is a case report.

## 3 Discussion

A definite history of trauma or iatrogenic injury can be found in cases of bladder ruptures. Cases of spontaneous ruptures of the urinary bladder without pathological injuries are clinically rare, with an incidence of 1:126,000, and a mortality of 47%.^[4]^

In this case, the patient had a history of binge drinking before the onset, and he was in a drunken state at the admission. There are 3 main reasons for high susceptibility for spontaneous bladder rupture after binge drinking.

Heavy drinking produces a diuretic effect, leading to an extreme filling of the bladder, increase in the tension of the bladder, and thinning of the bladder wall.

Alcohol intake will paralyze the central and peripheral nervous system and lead to bladder sensorium disorder, which inhibit the normal micturition reflex of the bladder.

Frequent nausea and vomiting in alcoholics increases the intraabdominal pressure and transmits this pressure to the weak bladder wall.^[5]^

Bladder ruptures can be divided into intraperitoneal and extraperitoneal types. Generally, the clinical symptoms of the former type are more serious, and resemble the symptoms of acute peritonitis. Thus, this disease can be easily confused with the acute abdomen triggered by other causes. Therefore, it is initially difficult to get an accurate diagnosis and requires an improved inspection and stricter clinical thought. Routine CT examination and abdominocentesis are essential. In this case, CT had clearly indicated the existence of intraperitoneal fluids, and abdominocentesis further confirmed the diagnosis. The immediate relief of abdominal pain after catheterization could be explained by the significantly reduced intraperitoneal extravasation of urine, intraperitoneal pressure, and peritoneal irritation after the relief of urinary retention. Considering this, we performed CT recheck within a short time and found significant absorption of the intraperitoneal fluids and disappearance of peritoneal irritation. These findings further confirmed that the acute abdomen in this patient was caused by extravasation of urine.^[6]^

Since cystography is theoretically the gold standard for the diagnosis of bladder rupture, we also performed this examination. Unfortunately, we did not capture the typical imaging findings of bladder rupture. It is possible that the bladder rupture was small, and since only 400 mL of contrast agent was used, it was impossible to extremely fill the bladder in case of secondary iatrogenic injury. Therefore, it is impossible to duplicate the extremely filled bladder under the drunken state.

Although spontaneous bladder rupture is partly due to the extremely filled bladder after binge drinking, we also need to exclude the possibility of lesions of the bladder itself. For cases like this one, lesions of the bladder, which need to be excluded, usually include the following: bladder outlet obstructions such as benign prostatic hyperplasia, cancer, urinary bladder stones, bladder blood clots, and urethral stricture, since urinary retention will cause extreme swelling of the bladder; bladder wall lesions such as cancer, tuberculosis, chronic inflammation, and bladder diverticulum; and neurogenic bladder. Therefore, a cystoscopy with biopsy is essential. During cystoscopy for our patient, we first excluded bladder outlet obstructions such as prostatic hyperplasia and urinary bladder stones, and found several, scattered, strawberry-like bulges associated with surface ulcers, and edematous thickening of the surrounding tissues at the bottom and top of the bladder. We then performed transurethral resection of the bladder, and the postoperative pathological examination indicated eosinophilic cystitis. Cystoscopy indicated that the patient had bladder lesions, which made him more susceptible to spontaneous bladder rupture.

Cases of eosinophilic cystitis are rare in clinical practice, and there are 200 clinical reports currently available.^[7]^ Moreover, there is only one report each from France and Korea of spontaneous bladder rupture caused by eosinophilic cystitis.^[2,8]^

At present, the cause of eosinophilic cystitis is not entirely clear, but it is generally considered to be associated with allergies, bladder cancer, and trauma. Various factors can cause cellular production of cytokines, which will elicit immune responses and eosinophil accumulations to start inflammatory reactions.^[9]^ Symptoms of eosinophilic cystitis could be mostly chronic lower urinary tract irritation, and in the late period they may manifest mainly as hematuria, dysuria, or urinary retention. Auxiliary examinations such as B-type ultrasound or CT could reveal changes like thickened bladder wall and masses in the bladder. The gold diagnosis standard is cystoscopy with biopsy, which can reveal masses in the bladder and surrounding mucosal edema and thickening. Histopathology mainly shows inflammatory lesions with several eosinophilic infiltrations.^[10]^

Eosinophilic cystitis is usually treated with conservative therapy. Patients with secondary eosinophilic cystitis caused by definite allergens can be cured by eliminating the predisposing factors. For patients with indefinite causes, long-term use of nonsteroidal antiinflammatory drugs and antihistamines is efficacious. Patients unresponsive to the above drugs can usually be cured by corticosteroids. Intravesical drugs can also be effective. Transurethral resection of bladder tumor usually has good prognosis, as few patients require partial or radical cystectomy. Since the causes of eosinophilic cystitis are unknown, a long-term, close follow-up of such patients is required, and routine cystoscopy with biopsy is also essential during follow-up.^[11]^

Our patient did not have a history of definite allergy, but a long-term history of alcohol abuse. Since eosinophilic cystitis is associated with long-term alcohol consumption, we recommended that the patient should stop drinking and taking antihistamines. This patient was followed up for 1 year, and the cystoscopy recheck showed that the bladder lesion had healed.

Cases of spontaneous bladder ruptures are rare, and those caused by a combination of binge drinking and eosinophilic cystitis are even rarer. Thus, the real relation of binge drinking and spontaneous bladder ruptures is still unknown, we plan to investigate it in future study.

## Acknowledgement

The authors thank grants from National Natural Science Foundation of China (81470919 and 81670632) for the support.
